# Coronary Artery Calcification Seen Through Chest Radiography

**DOI:** 10.14740/jocmr2121w

**Published:** 2015-07-24

**Authors:** Precil D. M. M. Neves, Ramaiane A. Bridi, Rosilene M. Elias, Rosa M. A. Moyses

**Affiliations:** a Nephrology Division, University of Sao Paulo, Sao Paulo, SP, Brazil

**Keywords:** End-stage renal disease, Coronary artery calcification, Hemodialysis, Chest radiography

## Abstract

Patients with end-stage renal disease (ESRD) on dialysis have poor overall survival, and cardiovascular (CV) is the main cause of mortality among these patients. Coronary calcification is an independent predictor of mortality and CV events in dialysis patients and can be accessed by using a computerized tomography scanning. The high cost of this procedure, however, precludes routine implementation of this method for the purposes of risk stratification. Aortic arch calcification has been associated with CV mortality in the general population. Also, vascular calcification beyond the thoracic aorta has been shown to be associated with mortality in ESRD patients. We presented here a case of a young patient with ESRD in which the coronary calcification could be cleared seen through simple chest radiography. This is a 35-year-old man with a history of ESRD secondary to pyelonephritis, who was receiving conventional hemodialysis thrice a week for the last 5 years. He was submitted to chest radiography as part of routine annual cardiac screening. His blood pressure was within the target limits, although much higher in lower limbs, generating a high ankle brachial index of 1.3. He also had secondary hyperparathyroidism. His physical examination was unremarkable, except for the presence of non-functioning arteriovenous fistulas in both arms and a central venous catheter. The last routine blood test showed calcium 9.0 mg/dL, phosphate 5.7 mg/dL, potassium 4.7 mEq/L, creatinine 7.4 mg/dL, alkaline phosphatase 175 U/L, and parathyroid hormone 1,745 pg/mL. Surprisingly, the chest radiography revealed a calcified aortic valve and a calcified coronary artery. This patient had sudden cardiac death few months after this radiography had been taken. We present here a case of coronary calcification that can be seen through simple chest radiography. Such images are not usually seen, although the risk of vascular calcification is high in this population, and is closely related to CV risk. Chest radiographs, nearly universally available provide a method for assessing coronary artery calcification. Such a finding is intriguing and should alert nephrologists and cardiologists for the high risk of CV death in these patients.

## Introduction

Patients with end-stage renal disease (ESRD) on dialysis have poor overall survival, with an age and sex adjusted mortality higher than patients not on dialysis [1]. Cardiovascular (CV) is the main cause of mortality among these patients [2]. Coronary calcification is an independent predictor of mortality and CV events in dialysis patients and can be accessed by using a computerized tomography scanning [3]. The high cost of computerized tomography scanning, however, precludes routine implementation of this method.

Bone and mineral metabolism factors, especially hyperparathyroidism are strongly associated with CV mortality in ESRD patients. Patients with secondary hyperparathyroidism and on dialysis are more likely to die of CV disease than the general population. In this field, other specific players such as fibroblast growth factor 23 (FGF-23) and sclerostin were already implicated in the pathogenesis and progression of coronary artery and aortic valve calcification in ESRD patients [4, 5].

Aortic arch calcification has been associated with CV mortality in the general population [6]. Also, vascular calcification beyond the thoracic aorta has been shown to be associated with mortality in ESRD patients [7].

## Case Report

**Figure 1:**
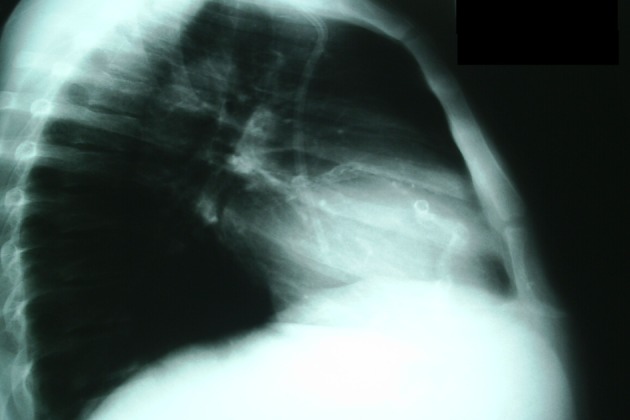
Chest radiography showing calcified right coronary and aortic valve.

This is a 35-year-old man with a history of ESRD who was receiving conventional hemodialysis for the last 5 years. He was submitted to chest radiography as part of routine annual cardiac screening. As associated comorbidity he had hypertension which has been treated with atenolol, atensin and amlodipine. His blood pressure was within the target limits, although much higher in lower limbs, generating a high ankle brachial index (ABI) of 1.3. He also had secondary hyperparathyroidism. The last routine blood test showed calcium 9.0 mg/dL, phosphate 5.7 mg/dL, potassium 4.7 mEq/L, creatinine 7.4 mg/dL, alkaline phosphatase 175 U/L, and parathyroid hormone 1,745 pg/mL. The chest radiography revealed a tunneled venous catheter, and incidentally found demarcated images on cardiac area (Fig. 1). The necklace shape extends from the cardiac apex to the base. A small circular shape is also displayed, superimposed on the first one. The lesions seen in this image correspond to a calcified right coronary and aortic valve. Parathyroidectomy (PTX) was indicated, but unfortunately, this patient had sudden cardiac death few months after this radiography had been taken. FGF-23, accessed afterward, was 1,327 pg/mL.

## Discussion

The presented patient had a high ABI, which is related to vessel wall stiffness and is not uncommon in dialysis patients. Low ABI is classically associated with peripheral artery disease and mortality. However, both low and high ABI can predict mortality in ESRD patients [8, 9]. The calcification seen in chest X-ray may be present in lower limbs vessels as well, explaining the high ABI in this patient.

Also, the secondary hyperparathyroidism may be contributed to the coronary artery calcification. Since FGF-23 is emerging as a new mortality marker, the high levels presented here might explain the unfavorable outcome in this case. The high dialysate calcium (3.5 mEq/L) as well as the calcium-based phosphate binder could worsen the coronary calcification and contribute to death [10]. Coronary calcification has not definitively linked to severity of hyperparathyroidism [11]. However, if high ABI and high levels of FGF-23 are present, the PTX should be performed as soon as possible, trying to reduce the sudden cardiac death risk associated to coronary calcification.

In summary, chest radiograph, nearly universally available, is inexpensive, and provides a method for assessing coronary artery calcification. This simple imaging modality might represent an easy assessment for coronary calcification in hemodialysis patients. Patients with hyperparathyroidism who presented high FGF-23 levels are at the high risk for vascular calcification and mortality. Nephrologists should seriously consider PTX treatment of hyperparathyroidism in ESRD patients on dialysis under the same clinical conditions, in order to avoid poor outcomes, as has been described here.
